# Impaired Cerebrovascular Reactivity in Huntington’s Disease

**DOI:** 10.3389/fphys.2021.663898

**Published:** 2021-07-21

**Authors:** Suk Tak Chan, Nathaniel D. Mercaldo, Kenneth K. Kwong, Steven M. Hersch, Herminia D. Rosas

**Affiliations:** ^1^Department of Radiology, Athinoula A. Martinos Center for Biomedical Imaging, Massachusetts General Hospital, Harvard Medical School, Charlestown, MA, United States; ^2^Department of Neurology, Massachusetts General Hospital, Harvard Medical School, Boston, MA, United States

**Keywords:** cerebrovascular reactivity, exogenous CO_2_ challenge, Huntington’s disease, functional magnetic resonance imaging, presymptomatic, perivascular space

## Abstract

There is increasing evidence that impairments of cerebrovascular function and/or abnormalities of the cerebral vasculature might contribute to early neuronal cell loss in Huntington’s disease (HD). Studies in both healthy individuals as well as in patients with other neurodegenerative disorders have used an exogenous carbon dioxide (CO_2_) challenge in conjunction with functional magnetic resonance imaging (fMRI) to assess regional cerebrovascular reactivity (CVR). In this study, we explored potential impairments of CVR in HD. Twelve gene expanded HD individuals, including both pre-symptomatic and early symptomatic HD and eleven healthy controls were administered a gas mixture targeting a 4–8 mmHg increase in CO_2_ relative to the end-tidal partial pressure of CO_2_ (P_*ET*_CO_2_) at rest. A Hilbert Transform analysis was used to compute the cross-correlation between the time series of regional BOLD signal changes (ΔBOLD) and increased P_*ET*_CO_2_, and to estimate the response delay of ΔBOLD relative to P_*ET*_CO_2_. After correcting for age, we found that the cross-correlation between the time series for regional ΔBOLD and for P_*ET*_CO_2_ was weaker in HD subjects than in controls in several subcortical white matter regions, including the corpus callosum, subcortical white matter adjacent to rostral and caudal anterior cingulate, rostral and caudal middle frontal, insular, middle temporal, and posterior cingulate areas. In addition, greater volume of dilated perivascular space (PVS) was observed to overlap, primarily along the periphery, with the areas that showed greater ΔBOLD response delay. Our preliminary findings support that alterations in cerebrovascular function occur in HD and may be an important, not as yet considered, contributor to early neuropathology in HD.

## Introduction

Huntington’s Disease (HD) is a devastating fully penetrant autosomal dominant progressive, rare neurological disorder that is characterized by progressive motor dysfunction, emotional disturbances, dementia, and weight loss (Conneally, 1984; Hersch and Rosas, 2008). It is caused by the abnormal expansion of a CAG repeat length in the gene that codes for an ubiquitously expressed protein, huntingtin (mHtt) (MacDonald et al., 1993), which can be found in neurons as well as all major components of the neurovascular unit including the basal lamina, endothelial cell, pericytes, smooth muscle cells (Lin et al., 2013; Drouin-Ouellet et al., 2015), astrocytes, and oligodendrocytes and microglia (Jansen et al., 2017). Chronic mHtt expression has been shown to alter the neurovasculature in an HD transgenic mouse model by upregulating wingless-related integration site (WNT) signaling activity and consequently inhibiting proper endothelial cell differentiation and maturation in the brain (Lim et al., 2017). The upregulation of WNT signaling activity in HD endothelial cells appeared to induce an increase in pericyte number (Stapor et al., 2014) and occurred early in the disease, leading to blood vessel sprouting, elongation, and early maturation. By late stage of disease, prominent aberrant vasculature was present (Padel et al., 2018). Alterations in the neurovasculature could result in increased cerebral blood volume, reduced cerebral blood flow (CBF), increased small vessel density and increased blood-brain barrier (BBB) permeability, as has reported in rodent models of HD and patient post-mortem tissue (Chen et al., 2012; Franciosi et al., 2012; Lin et al., 2013; Hua et al., 2014; Drouin-Ouellet et al., 2015; Hsiao et al., 2015). These studies provided early support for neurovascular dysfunction in HD. Impairments in neurovascular function have also been associated with enlarged perivascular spaces (PVS), which are considered not only a hallmark of small vessel disease (Brown et al., 2018), but of perivascular inflammation and impaired clearance of proteins (Brown et al., 2018). Using a novel automated segmentation algorithm (Chan et al., 2020b), we found that the load of enlarged PVS was greater in patients with HD in a manner that directly correlated with disease severity and caudate atrophy. Together, these findings converge to provide support for early impairment of cerebrovascular function, including prior to neuronal cell loss or to early clinical symptoms including weight loss, behavioral changes (Padel et al., 2018) or motor dysfunction (Lin et al., 2013; Drouin-Ouellet et al., 2015) in HD.

To date, studies have not as yet evaluated early cerebrovascular dysfunction *in vivo* in HD. In particular, mild physiological stress can be used to depict subtle vascular abnormalities prior to the failure in autoregulation. It is possible that early cerebrovascular dysfunction could contribute to early neuropathological changes in HD. In this study, we used hypercapnic stress, administering an air mixture with a low concentration of carbon dioxide (CO_2_) as a mild physiological stress, to probe regional cerebrovascular reactivity (CVR) in HD gene-expanded individuals, both motor pre-symptomatic and early symptomatic individuals. We mapped regional cerebrovascular responses to the CO_2_ challenge using blood oxygenation level dependent (BOLD) signal changes measured with functional magnetic resonance imaging (fMRI). This approach has a higher temporal resolution and higher contrast to noise ratio than arterial spin labeling (ASL), and might therefore be better able to more precisely demonstrate the vascular responses in white matter (Schmithorst et al., 2014). The extent of regional impairment in CVR was evaluated using the cross-correlation between regional BOLD signal changes and increased CO_2_ level, indicated by end-tidal partial pressure of CO_2_ (P_*ET*_CO_2_), using a Hilbert Transform analysis (Saad et al., 2003). A correlation was also employed with the total functional capacity (TFC), a measure of clinical severity, to explore the potential clinical impact associated with impaired cerebrovascular function. Our findings support that early changes in CVR occur in HD and support the importance of considering neurovascular alterations as a key pathophysiological mechanism that could both contribute to subclinical progression and could also serve as a novel target for intervention.

## Materials and Methods

### Participants

Huntington’s disease gene-expanded individuals, both motor pre-symptomatic and early symptomatic, were recruited from the Center of Excellence in the Department of Neurology at Massachusetts General Hospital. Healthy volunteers were recruited through the Partners hospital network and were screened to exclude neurological, mental and medical disorders, drug abuse, and contraindications for exposure to high magnetic field. Clinical assessments were conducted by an HD specialist (HDR). All MRI scanning was performed at the Athinoula A. Martinos Center for Biomedical Imaging at the Massachusetts General Hospital. All the experimental procedures were explained to the subjects. Signed informed consent was obtained before any study procedures were conducted. All components of this study were performed in compliance with the Declaration of Helsinki and all procedures were approved by the MGH Human Research Committee (IRB Protocol Number: 2014P002240).

### MRI Acquisition

Magnetic resonance imaging brain scanning was performed on a 3-Tesla scanner (Siemens Medical, Erlangen, Germany). The head was immobilized in a standard head coil with foam pads. The following whole brain MRI datasets were acquired: (1) standard high-resolution 3D sagittal images acquired with volumetric T1-weighted 3D-MEMPRAGE sequence (TR = 2530 ms, TE = 1.74, 3.6, 5.46, and 7.32 ms, flip angle = 7°, FOV = 256 × 256 mm, matrix = 256 × 256, slice thickness = 1 mm); (2) T2-SPACE (TR = 3200 ms, TE = 454, flip angle = 120°, FOV = 256 × 256 mm, matrix = 256 × 256, slice thickness = 1 mm); and (3) BOLD-fMRI images acquired with gradient-echo echo planar imaging (EPI) sequence (TR = 1250 ms, TE = 30 ms, flip angle = 90°, FOV = 220 × 220 mm, matrix = 64 × 64, thickness = 5 mm, slice ga*p* = 1 mm) during the hypercapnic challenge.

### Hypercapnic Challenge With Externally Administered CO_2_

**FIGURE 1:**
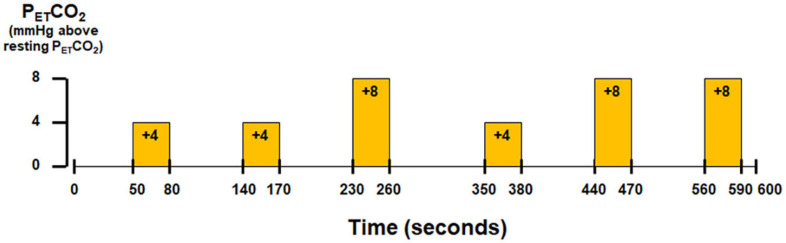
Paradigm of hypercapnic challenge using externally administered CO_2_.

Subjects wore a nose-clip and breathed through a mouth-piece using an MRI-compatible circuit designed to maintain the P_*ET*_CO_2_ within ±1–2 mmHg of the target P_*ET*_CO_2_ (Banzett et al., 2000; McKay et al., 2003). Given the potential for inter-individual variance in the resting end-tidal partial pressure of CO_2_ (P_*ET*_CO_2_) (West, 1992), the resting P_*ET*_CO_2_ was assessed for each individual subject via calibrated capnography before the hypercapnic challenge. The fraction of inspired carbon dioxide was adjusted to produce steady-state conditions of normocapnia and mild hypercapnia (4–8 mmHg above the subject’s resting P_*ET*_CO_2_). The CO_2_ challenge paradigm consisted of 2 consecutive phases (normocapnia and mild hypercapnia) repeating six times with three epochs of 4 mmHg increase and three epochs of 8 mmHg increase of P_*ET*_CO_2_ (Figure 1). The normocapnia phase lasted 60–90 s, while the mild hypercapnia phase lasted 30 s. The total duration of the hypercapnic challenge lasted 10 min.

### Physiological Recording

Physiological changes, including the partial pressure of carbon dioxide (PCO_2_) and oxygen (PO_2_), and respiratory flow, were measured simultaneously with the MRI acquisition. PCO_2_ and PO_2_ were sampled through an air filter connected to the mouthpiece and the sampled gases were measured using gas analyzers (Capstar-100, Oxystar-100, CWE, Inc., PA, United States), after calibrating to the barometric pressure of the day of MRI scanning and correcting for vapor pressure. Respiratory flow was measured using a respiratory flow head (MTL300L, ADInstruments, Inc., CO, United States) on a breathing circuit via calibrated spirometer (FE141, ADInstruments, Inc., CO, United States). The recording of PCO_2_, PO_2_, respiratory flow was integrated into Powerlab and LabChart (ADInstruments, Inc., CO, United States). The physiological measurements were synchronized with the MRI images using trigger signals from the scanner. The BOLD-fMRI images and physiological recordings were stored for off-line data analysis.

### Data Analysis

#### Processing of Physiological Data

Physiological data were analyzed using Matlab R2014a (Mathworks, Inc., Natick, MA, United States). Technical delays of PCO_2_ and PO_2_ were corrected by cross-correlating the time series of PCO_2_ and PO_2_ with the respiratory flow tracing acquired with the spirometer. End inspiration (I) and end expiration (E) were defined on the time series of PO_2_ and PCO_2_ and were verified by the inspiratory and expiratory phases of the respiratory flow time series. The breath-to-breath P_*ET*_CO_2_ and end-tidal O_2_ (P_*ET*_O_2_) were extracted at the end expiration of PCO_2_ and PO_2_ time series, respectively.

#### Cross-Correlation Analysis Between BOLD-fMRI Data and P_*ET*_CO_2_

The BOLD-fMRI data were imported into the Analysis of Functional NeuroImage (AFNI) (Cox, 1996) (National Institute of Mental Health)^1^ software for data analysis. Details of preprocessing with AFNI are given as follows. The first 12 volumes of each functional dataset that were collected before equilibrium magnetization was reached were discarded. Each functional dataset was corrected for slice timing, motion-corrected and co-registered to the first image of the first functional dataset using a three-dimensional volume registration. In the co-registered functional dataset, the time series of each voxel was de-trended with the fifth order of polynomials to remove the low drift frequency, and the components of motion were removed using orthogonal projection. The clean functional dataset was then normalized to its mean intensity value across the time series. Voxels located within the ventricles and outside the brain defined in the parcellated brain volume using FreeSurfer version 5.3 (Dale et al., 1999; Fischl et al., 1999) (MGH/MIT/HMS Athinoula A. Martinos Center for Biomedical Imaging, Boston)^2^ were excluded. Individual subject brain volumes with time series of percent BOLD signal changes (ΔBOLD) were derived. Taking advantage of Fast Fourier transforms, a Hilbert Transform analysis (Saad et al., 2003) was used to measure the cross-correlation between the ΔBOLD and the reference time series of P_*ET*_CO_2_ in each voxel and the associated delay of BOLD response relative to the changes in P_*ET*_CO_2_.

The statistical parametric maps for individual subjects were cluster-corrected using a threshold estimated with a Monte Carlo simulation algorithm. Individual subject brain volumes with cross-correlation coefficients were registered onto each subject’s anatomical scan and transformed to the standardized space of Talairach and Tournoux (Talairach and Tournoux, 1988). In order to protect against type I error, an individual voxel probability threshold of *p* < 0.005 was held to correct the overall significance level to α < 0.05. Monte Carlo simulation was used to correct for multiple comparisons (Gold et al., 1998). Based upon a Monte Carlo simulation, using 2000 iteration processed with ClustSim program (Ward, 1997), it was estimated that a 226 mm^3^ contiguous volume would provide the overall corrected threshold of *p* < 0.05.

#### Group Analysis of Demographics, CVR, and Disease Severity Between Patients and Controls

Descriptive summaries were computed by diagnostic group. Categorical variables were summarized as frequencies and percentages and group differences were assessed using either the Chi-square test or Fisher’s exact test. Continuous variables were summarized as median (interquartile range = 25th and 75th percentiles) and group differences were assessed using Kruskal-Wallis test.

Cross-correlation coefficients in individual subject maps were transformed to z-scores for the group analysis (z = ln[(1 + r)/(1-r)]/2) (Fisher, 1921). For each subject, these z-scores were averaged in each of the 160 brain regions parcellated using FreeSurfer version 5.3 (Dale et al., 1999; Fischl et al., 1999). A linear regression model was constructed to quantify the association between CVR, as indicated by the average z-score, and disease designation (HD and control) after adjusting for age. Separate models were estimated for each region. For each model, linear combinations of parameter estimates were computed to estimate average z-values by group (fixing age = 50), and their associated 95% confidence intervals. Differences in average z-scores (HD-control) were also estimated, after adjusting for age, and the 95% confidence and unadjusted p-value.

Partial spearman correlation coefficients (Liu et al., 2018) were estimated to summarize the monotonic relationship between *z*-scores and TFC for all subjects after adjusting for age. False discovery rate adjusted p-values were computed to account for multiple comparisons (Benjamini and Hochberg, 1995). All group analyses were performed using R 4.0.2^3^.

## Results

Twenty-three participants (14 males, 9 females, aged from 22 to 62 years) were included. Eleven were healthy volunteers (median = 32.0 years, interquartile range IQR = 27.5–46.0 years; 9M and 2F) and the remaining 12 were HD gene-expanded individuals (median = 51.5 years, interquartile range IQR = 47.3–57.3 years; 5M and 7F), including both pre-symptomatic and early symptomatic HD subjects. The selected clinical characteristics of the gene-expanded subjects are shown in Table 1. Significant differences were observed between HD and control groups in terms of age (*p* = 0.026) but marginally for gender (*p* = 0.123).

**TABLE 1:** Subject demographics.

	**Control**	**HD**	***p***
N	11	12	
Age	32.00 [27.50, 46.00]	51.50 [47.25, 57.25]	0.026*
Sex: Male	9 (81.8)	5 (41.7)	0.123
TFC	–	13 [11, 13]	

Categorical variable (i.e., sex) is summarized as frequency (percentage) and continuous variable (i.e., age) is summarized as median [interquartile range]. TFC, total functional capacity. *significant difference *p* < 0.05.

### Group Comparison of Cross-Correlation of ΔBOLD With P_*ET*_CO_2_ Between HD Gene-Expanded Subjects and Controls

**FIGURE 2:**
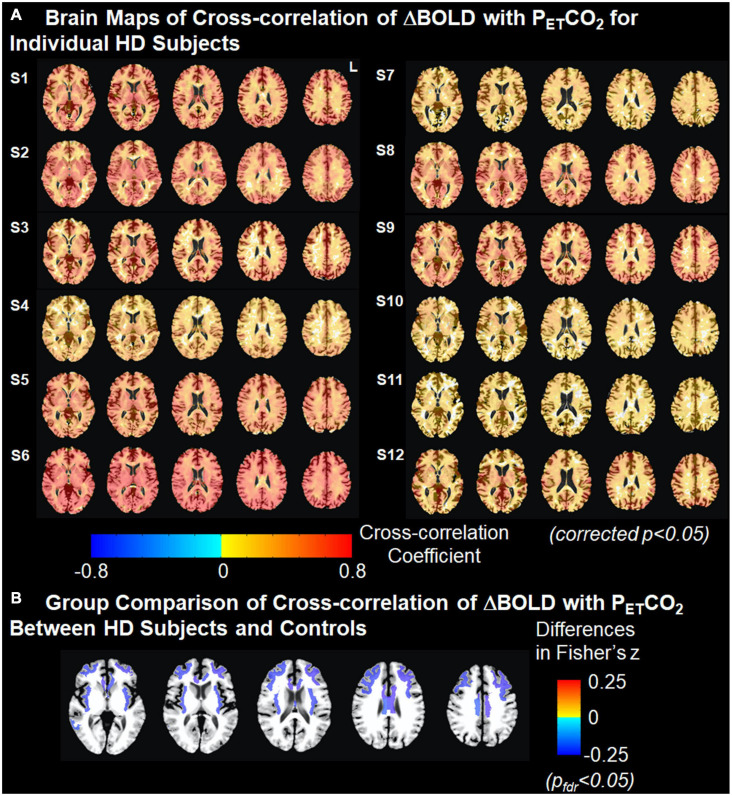
Cross-correlation of ΔBOLD with P_*ET*_CO_2_. **(A)** Brain maps of cross-correlation of ΔBOLD with P_*ET*_CO_2_ for individual HD subjects. The strength of cross-correlation increases from light color to dark color. Brain regions without color indicate that the ΔBOLD in these areas are not correlated with the changes in P_*ET*_CO_2_. Multiple comparisons are corrected using clustering at an overall *p* < 0.05. **(B)** Group comparison of Fisher-transformed cross-correlation values between HD subjects and controls after adjusting for age. Cold colors represent the weaker cross-correlation found in HD subjects than in controls. Multiple comparisons were corrected at false discovery rate *p*_*fdr*_ < 0.05.

Brain maps with the cross-correlation of ΔBOLD with P_*ET*_CO_2_ superimposed on the T1 image and the associated ΔBOLD response delay superimposed on the T2-T1 ratio image for individual gene-expanded subjects are shown in Figure 2 and Supplementary Figure 1, respectively.

After correcting for age, we found that the cross-correlation between the time series for regional ΔBOLD and for P_*ET*_CO_2_ in several subcortical white matter regions was significantly weaker in gene-expanded subjects than in controls (Figure 2 and Table 2). Regions of significance included corpus callosum (mid-posterior segment: HD-Control estimate = −0.23, 95% CI = −0.37, −0.08, *p* = 0.002, *p*_*fdr*_ = 0.041; central segment: HD-Control estimate = −0.24, 95% CI = −0.38, −0.11, *p* = 0.001, *p*_*fdr*_ < 0.029), subcortical white matter adjacent to rostral and caudal anterior cingulate, rostral and caudal middle frontal, insular, middle temporal, and posterior cingulate areas. Greater dilated PVS load was also observed to overlap with and along the periphery of the areas that showed greater ΔBOLD response delays (Supplementary Figure 1). In the symptomatic HD group, no significant correlation was found between TFC and the *z*-scores indicating the cross correlation of ΔBOLD with P_*ET*_CO_2_ after adjusting for age (*p*_*fdr*_ > 0.05).

**TABLE 2:** Association between Fisher-transformed cross-correlation values and disease designation (HD vs. Control) after adjusting for age.

**Corpus callosum**	**Control**		**HD**		**HD-Control**			**Unadjusted**
	**Estimate (95% CI)**		**Estimate (95% CI)**		**Estimate (95% CI)**			***p*-value**
Posterior segment	0.44 (0.34,0.55)		0.40 (0.33,0.48)		−0.04 (−0.17, 0.09)			0.560
Midposterior segment	0.52 (0.41,0.64)		0.30 (0.22,0.38)		−0.23 (−0.37, −0.08)			0.002*
Central segment	0.53 (0.42,0.64)		0.28 (0.20,0.36)		−0.24 (−0.38, −0.11)			0.001*
Midanterior segment	0.53 (0.38,0.67)		0.32 (0.21,0.42)		−0.21 (−0.39, −0.03)			0.020
Anterior segment	0.56 (0.41,0.71)		0.32 (0.22,0.43)		−0.24 (−0.42, −0.06)			0.010

**Left Hemisphere**					**Right Hemisphere**			

**Brain regions**	**Control Estimate (95% CI)**	**HD Estimate (95% CI)**	**HD-Control Estimate (95% CI)**	**Unadjusted *p*-value**	**Control Estimate (95% CI)**	**HD Estimate (95% CI)**	**HD-Control Estimate (95% CI)**	**Unadjusted *p*-value**

Thalamus	0.59 (0.42, 0.75)	0.57 (0.45, 0.69)	−0.02 (−0.23, 0.19)	0.858	0.58 (0.41, 0.74)	0.56 (0.45, 0.68)	−0.01 (−0.22, 0.19)	0.904
Caudate	0.55 (0.38, 0.71)	0.51 (0.39, 0.62)	−0.04 (−0.24, 0.16)	0.696	0.56 (0.39, 0.72)	0.52 (0.40, 0.64)	−0.04 (−0.24, 0.16)	0.706
Putamen	0.54 (0.36, 0.71)	0.52 (0.39, 0.65)	−0.02 (−0.24, 0.20)	0.866	0.57 (0.40, 0.74)	0.52 (0.40, 0.64)	−0.05 (−0.26, 0.16)	0.631
Pallidum	0.55 (0.43, 0.66)	0.37 (0.29, 0.46)	−0.18 (−0.32, −0.03)	0.017	0.54 (0.42, 0.66)	0.37 (0.28, 0.45)	−0.18 (−0.33, −0.03)	0.022
Hippocampus	0.63 (0.49, 0.76)	0.47 (0.37, 0.56)	−0.16 (−0.33, 0.01)	0.057	0.64 (0.50, 0.77)	0.45 (0.35, 0.54)	−0.19 (−0.36, −0.02)	0.027
Amygdala	0.59 (0.45, 0.73)	0.41 (0.30, 0.51)	−0.18 (−0.36, −0.01)	0.042	0.55 (0.42, 0.69)	0.38 (0.29, 0.48)	−0.17 (−0.34, 0.00)	0.045
Accumbens area	0.45 (0.30, 0.59)	0.39 (0.28, 0.50)	−0.05 (−0.24, 0.13)	0.554	0.46 (0.33, 0.58)	0.35 (0.25, 0.44)	−0.11 (−0.27, 0.05)	0.164
Banks of superior temporal sulcus	0.68 (0.50, 0.86)	0.57 (0.44, 0.70)	−0.11 (−0.33, 0.12)	0.343	0.69 (0.51, 0.87)	0.57 (0.44, 0.70)	−0.12 (−0.34, 0.10)	0.285
Caudal anterior cingulate	0.63 (0.47, 0.79)	0.63 (0.52, 0.75)	0.00 (−0.19, 0.20)	0.969	0.63 (0.47, 0.79)	0.61 (0.50, 0.73)	−0.02 (−0.22, 0.18)	0.856
Caudal middle frontal	0.71 (0.54, 0.87)	0.58 (0.46, 0.70)	−0.13 (−0.33, 0.08)	0.220	0.67 (0.50, 0.85)	0.60 (0.47, 0.73)	−0.07 (−0.29, 0.15)	0.513
Cuneus	0.65 (0.45, 0.86)	0.61 (0.46, 0.76)	−0.04 (−0.30, 0.21)	0.741	0.61 (0.41, 0.81)	0.60 (0.45, 0.75)	−0.01 (−0.26, 0.24)	0.918
Entorhinal	0.39 (0.30, 0.48)	0.31 (0.24, 0.37)	−0.09 (−0.20, 0.03)	0.138	0.35 (0.25, 0.46)	0.32 (0.25, 0.40)	−0.03 (−0.16, 0.10)	0.657
Fusiform	0.55 (0.39, 0.71)	0.56 (0.44, 0.68)	0.01 (−0.19, 0.21)	0.905	0.54 (0.39, 0.69)	0.54 (0.43, 0.65)	0.00 (−0.18, 0.19)	0.985
Inferior parietal	0.71 (0.55, 0.87)	0.55 (0.43, 0.67)	−0.16 (−0.36, 0.04)	0.127	0.70 (0.53, 0.86)	0.55 (0.43, 0.67)	−0.15 (−0.36, 0.06)	0.163
Inferior temporal	0.45 (0.34, 0.56)	0.43 (0.35, 0.51)	−0.02 (−0.16, 0.11)	0.748	0.45 (0.33, 0.56)	0.43 (0.35, 0.52)	−0.02 (−0.16, 0.13)	0.834
Isthmus of cingulate	0.55 (0.37, 0.72)	0.58 (0.46, 0.71)	0.04 (−0.18, 0.25)	0.734	0.56 (0.39, 0.73)	0.59 (0.46, 0.71)	0.03 (−0.18, 0.24)	0.809
Lateral occipital	0.63 (0.47, 0.80)	0.55 (0.44, 0.67)	−0.08 (−0.28, 0.12)	0.436	0.62 (0.46, 0.78)	0.58 (0.46, 0.69)	−0.05 (−0.24, 0.15)	0.648
Lateral orbitofrontal	0.45 (0.33, 0.57)	0.38 (0.30, 0.47)	−0.07 (−0.21, 0.08)	0.355	0.43 (0.32, 0.54)	0.41 (0.33, 0.49)	−0.02 (−0.16, 0.12)	0.783
Lingual	0.57 (0.38, 0.76)	0.62 (0.48, 0.75)	0.04 (−0.19, 0.28)	0.725	0.57 (0.38, 0.76)	0.60 (0.46, 0.74)	0.04 (−0.20, 0.27)	0.759
Medial orbitofrontal	0.46 (0.36, 0.56)	0.34 (0.27, 0.42)	−0.12 (−0.24, 0.01)	0.060	0.45 (0.35, 0.54)	0.35 (0.29, 0.42)	−0.09 (−0.21, 0.02)	0.118
Middle temporal	0.57 (0.43, 0.70)	0.48 (0.38, 0.57)	−0.09 (−0.25, 0.08)	0.287	0.58 (0.45, 0.70)	0.48 (0.39, 0.58)	−0.09 (−0.25, 0.06)	0.237
Parahippocampal	0.55 (0.42, 0.68)	0.47 (0.37, 0.56)	−0.09 (−0.25, 0.08)	0.306	0.54 (0.40, 0.69)	0.44 (0.33, 0.54)	−0.11 (−0.29, 0.07)	0.248
Paracentral	0.64 (0.45, 0.83)	0.66 (0.52, 0.80)	0.02 (−0.22, 0.26)	0.867	0.63 (0.45, 0.81)	0.67 (0.53, 0.80)	0.03 (−0.19, 0.26)	0.764
Pars opercularis	0.65 (0.47, 0.84)	0.61 (0.47, 0.74)	−0.05 (−0.27, 0.18)	0.676	0.63 (0.46, 0.80)	0.62 (0.50, 0.75)	−0.01 (−0.22, 0.20)	0.923
Pars orbitalis	0.51 (0.35, 0.67)	0.49 (0.37, 0.60)	−0.02 (−0.22, 0.18)	0.838	0.49 (0.34, 0.65)	0.54 (0.42, 0.65)	0.04 (−0.15, 0.23)	0.677
Pars triangularis	0.59 (0.42, 0.76)	0.54 (0.42, 0.67)	−0.04 (−0.25, 0.17)	0.684	0.58 (0.43, 0.74)	0.56 (0.45, 0.68)	−0.02 (−0.22, 0.18)	0.843
Peri-calcarine	0.62 (0.40, 0.84)	0.62 (0.46, 0.79)	0.00 (−0.28, 0.28)	0.984	0.60 (0.39, 0.81)	0.60 (0.44, 0.75)	0.00 (−0.26, 0.26)	0.983
Postcentral	0.67 (0.50, 0.84)	0.62 (0.50, 0.75)	−0.05 (−0.26, 0.17)	0.672	0.64 (0.47, 0.81)	0.62 (0.49, 0.74)	−0.03 (−0.23, 0.18)	0.800
Posterior cingulate	0.64 (0.46, 0.82)	0.61 (0.48, 0.74)	−0.03 (−0.25, 0.20)	0.815	0.61 (0.44, 0.78)	0.60 (0.48, 0.73)	−0.01 (−0.22, 0.21)	0.960
Precentral	0.68 (0.51, 0.84)	0.59 (0.47, 0.71)	−0.08 (−0.29, 0.12)	0.426	0.65 (0.48, 0.81)	0.60 (0.48, 0.72)	−0.04 (−0.25, 0.16)	0.661
Precuneus	0.63 (0.45, 0.81)	0.59 (0.46, 0.71)	−0.04 (−0.26, 0.17)	0.695	0.64 (0.46, 0.82)	0.58 (0.45, 0.71)	−0.06 (−0.28, 0.16)	0.602
Rostral anterior cingulate	0.57 (0.41, 0.73)	0.52 (0.41, 0.64)	−0.04 (−0.24, 0.16)	0.679	0.58 (0.44, 0.72)	0.50 (0.40, 0.60)	−0.08 (−0.25, 0.09)	0.357
Rostral middle frontal	0.65 (0.50, 0.81)	0.54 (0.43, 0.66)	−0.11 (−0.30, 0.08)	0.266	0.64 (0.48, 0.79)	0.56 (0.45, 0.67)	−0.08 (−0.26, 0.11)	0.419
Superior frontal	0.63 (0.47, 0.78)	0.58 (0.46, 0.69)	−0.05 (−0.25, 0.14)	0.602	0.63 (0.47, 0.80)	0.58 (0.46, 0.70)	−0.05 (−0.25, 0.15)	0.602
Superior parietal	0.68 (0.52, 0.84)	0.60 (0.48, 0.71)	−0.08 (−0.28, 0.11)	0.406	0.68 (0.52, 0.84)	0.59 (0.47, 0.71)	−0.09 (−0.29, 0.11)	0.361
Superior temporal	0.63 (0.48, 0.78)	0.52 (0.40, 0.63)	−0.11 (−0.30, 0.07)	0.238	0.62 (0.47, 0.78)	0.54 (0.42, 0.65)	−0.09 (−0.28, 0.11)	0.381
Supramarginal	0.69 (0.52, 0.86)	0.62 (0.50, 0.74)	−0.07 (−0.28, 0.14)	0.513	0.65 (0.48, 0.82)	0.62 (0.50, 0.74)	−0.03 (−0.24, 0.18)	0.765
Frontal pole	0.36 (0.25, 0.48)	0.28 (0.19, 0.36)	−0.09 (−0.23, 0.06)	0.243	0.41 (0.28, 0.54)	0.33 (0.23, 0.42)	−0.08 (−0.25, 0.08)	0.303
Transverse temporal	0.63 (0.42, 0.84)	0.61 (0.46, 0.77)	−0.02 (−0.27, 0.24)	0.908	0.62 (0.41, 0.82)	0.64 (0.49, 0.78)	0.02 (−0.23, 0.27)	0.873
Insula	0.63 (0.45, 0.82)	0.54 (0.41, 0.68)	−0.09 (−0.32, 0.14)	0.441	0.62 (0.45, 0.79)	0.56 (0.43, 0.68)	−0.06 (−0.27, 0.15)	0.570
Banks of superior temporal sulcus WM	0.61 (0.46, 0.76)	0.43 (0.32, 0.54)	−0.18 (−0.36, 0.01)	0.059	0.57 (0.44, 0.71)	0.40 (0.31, 0.50)	−0.17 (−0.33, −0.01)	0.042
Caudal anterior cingulate WM	0.65 (0.52, 0.78)	0.40 (0.31, 0.50)	−0.25 (−0.40, −0.09)	0.002*	0.66 (0.55, 0.78)	0.40 (0.31, 0.48)	−0.27 (−0.41, −0.12)	< 0.001*
Caudal middle frontal WM	0.63 (0.51, 0.75)	0.40 (0.31, 0.49)	−0.23 (−0.38, −0.07)	0.004*	0.64 (0.49, 0.78)	0.44 (0.33, 0.54)	−0.20 (−0.38, −0.02)	0.026
Cuneus WM	0.68 (0.50, 0.86)	0.59 (0.46, 0.72)	−0.10 (−0.32, 0.13)	0.397	0.67 (0.49, 0.84)	0.57 (0.44, 0.70)	−0.10 (−0.31, 0.12)	0.389
Entorhinal WM	0.47 (0.36, 0.57)	0.31 (0.23, 0.39)	−0.16 (−0.29, −0.03)	0.017	0.49 (0.38, 0.61)	0.31 (0.23, 0.40)	−0.18 (−0.33, −0.04)	0.014
Fusiform WM	0.59 (0.45, 0.73)	0.46 (0.35, 0.56)	−0.13 (−0.30, 0.04)	0.137	0.57 (0.44, 0.69)	0.45 (0.35, 0.54)	−0.12 (−0.28, 0.03)	0.125
Inferior parietal WM	0.64 (0.49, 0.78)	0.44 (0.34, 0.54)	−0.20 (−0.37, −0.02)	0.029	0.61 (0.48, 0.75)	0.42 (0.33, 0.52)	−0.19 (−0.36, −0.02)	0.026
Inferior temporal WM	0.52 (0.42, 0.63)	0.40 (0.32, 0.48)	−0.12 (−0.25, 0.01)	0.065	0.50 (0.40, 0.60)	0.40 (0.32, 0.47)	−0.10 (−0.23, 0.03)	0.119
Isthmus of cingulate WM	0.61 (0.49, 0.73)	0.44 (0.35, 0.53)	−0.17 (−0.32, −0.02)	0.025	0.60 (0.49, 0.72)	0.43 (0.34, 0.51)	−0.17 (−0.32, −0.03)	0.017
Lateral occipital WM	0.63 (0.48, 0.78)	0.49 (0.38, 0.60)	−0.14 (−0.32, 0.05)	0.139	0.61 (0.47, 0.74)	0.49 (0.39, 0.58)	−0.12 (−0.28, 0.05)	0.155
Lateral orbitofrontal WM	0.53 (0.42, 0.65)	0.35 (0.27, 0.43)	−0.18 (−0.32, −0.05)	0.009	0.50 (0.40, 0.60)	0.38 (0.31, 0.46)	−0.12 (−0.24, 0.01)	0.066
Lingual WM	0.61 (0.45, 0.78)	0.54 (0.42, 0.66)	−0.07 (−0.27, 0.13)	0.499	0.63 (0.47, 0.79)	0.52 (0.41, 0.64)	−0.11 (−0.30, 0.09)	0.284
Medial orbitofrontal WM	0.52 (0.42, 0.62)	0.35 (0.28, 0.42)	−0.17 (−0.30, −0.05)	0.006	0.51 (0.41, 0.61)	0.35 (0.27, 0.42)	−0.16 (−0.29, −0.04)	0.012
Middle temporal WM	0.63 (0.49, 0.77)	0.44 (0.34, 0.54)	−0.19 (−0.36, −0.02)	0.033	0.63 (0.52, 0.74)	0.42 (0.34, 0.50)	−0.21 (−0.35, −0.07)	0.003*
Parahippocampal WM	0.61 (0.48, 0.74)	0.44 (0.34, 0.53)	−0.17 (−0.34, −0.01)	0.033	0.61 (0.49, 0.73)	0.42 (0.33, 0.50)	−0.20 (−0.35, −0.05)	0.010
Paracentral WM	0.59 (0.44, 0.74)	0.48 (0.36, 0.59)	−0.12 (−0.31, 0.07)	0.231	0.53 (0.39, 0.67)	0.46 (0.36, 0.56)	−0.07 (−0.24, 0.10)	0.415
Pars opercularis WM	0.70 (0.56, 0.84)	0.47 (0.37, 0.58)	−0.22 (−0.40, −0.05)	0.012	0.67 (0.53, 0.82)	0.46 (0.35, 0.56)	−0.22 (−0.39, −0.04)	0.015
Pars orbitalis WM	0.59 (0.43, 0.75)	0.47 (0.35, 0.58)	−0.12 (−0.31, 0.08)	0.236	0.57 (0.42, 0.73)	0.51 (0.40, 0.62)	−0.06 (−0.25, 0.13)	0.533
Pars triangularis WM	0.63 (0.49, 0.78)	0.43 (0.33, 0.54)	−0.20 (−0.38, −0.03)	0.024	0.63 (0.50, 0.76)	0.45 (0.36, 0.55)	−0.18 (−0.34, −0.01)	0.033
Peri-calcarine WM	0.65 (0.48, 0.81)	0.54 (0.42, 0.66)	−0.10 (−0.31, 0.10)	0.327	0.63 (0.47, 0.79)	0.52 (0.41, 0.64)	−0.11 (−0.30, 0.09)	0.292
Postcentral WM	0.71 (0.55, 0.86)	0.55 (0.44, 0.67)	−0.16 (−0.35, 0.04)	0.112	0.67 (0.52, 0.82)	0.55 (0.44, 0.66)	−0.12 (−0.31, 0.07)	0.209
Posterior cingulate WM	0.65 (0.52, 0.77)	0.39 (0.30, 0.48)	−0.26 (−0.41, −0.10)	0.001*	0.60 (0.48, 0.71)	0.39 (0.31, 0.47)	−0.20 (−0.34, −0.07)	0.004*
Precentral WM	0.63 (0.50, 0.76)	0.43 (0.34, 0.53)	−0.20 (−0.36, −0.03)	0.019	0.60 (0.47, 0.73)	0.43 (0.34, 0.53)	−0.17 (−0.33, 0.00)	0.048
Precuneus WM	0.62 (0.49, 0.75)	0.46 (0.36, 0.56)	−0.16 (−0.32, 0.00)	0.056	0.59 (0.46, 0.73)	0.44 (0.35, 0.54)	−0.15 (−0.32, 0.02)	0.088
Rostral anterior cingulate WM	0.62 (0.50, 0.74)	0.39 (0.30, 0.48)	−0.23 (−0.39, −0.08)	0.003*	0.63 (0.51, 0.74)	0.36 (0.28, 0.45)	−0.26 (−0.40, −0.12)	<0.001*
Rostral middle frontal WM	0.66 (0.53, 0.79)	0.42 (0.32, 0.51)	−0.24 (−0.40, −0.08)	0.003*	0.66 (0.54, 0.78)	0.43 (0.35, 0.52)	−0.23 (−0.37, −0.08)	0.003*
Superior frontal WM	0.65 (0.51, 0.78)	0.45 (0.35, 0.55)	−0.20 (−0.37, −0.03)	0.024	0.63 (0.50, 0.76)	0.44 (0.35, 0.54)	−0.19 (−0.35, −0.02)	0.027
Superior parietal WM	0.63 (0.50, 0.76)	0.45 (0.36, 0.55)	−0.18 (−0.34, −0.02)	0.027	0.58 (0.45, 0.72)	0.46 (0.36, 0.56)	−0.13 (−0.30, 0.05)	0.149
Superior temporal WM	0.65 (0.52, 0.79)	0.43 (0.33, 0.52)	−0.23 (−0.39, −0.07)	0.006	0.68 (0.54, 0.82)	0.48 (0.37, 0.58)	−0.21 (−0.38, −0.03)	0.021
Supramarginal WM	0.68 (0.54, 0.82)	0.48 (0.38, 0.58)	−0.20 (−0.37, −0.03)	0.021	0.61 (0.48, 0.74)	0.46 (0.37, 0.56)	−0.15 (−0.31, 0.02)	0.084
Frontal pole WM	0.37 (0.25, 0.49)	0.31 (0.22, 0.40)	−0.06 (−0.21, 0.09)	0.453	0.45 (0.29, 0.60)	0.40 (0.28, 0.51)	−0.05 (−0.24, 0.14)	0.600
Transverse temporal WM	0.69 (0.52, 0.86)	0.52 (0.40, 0.65)	−0.17 (−0.38, 0.04)	0.119	0.66 (0.49, 0.82)	0.56 (0.44, 0.68)	−0.10 (−0.31, 0.11)	0.356
Insula WM	0.67 (0.55, 0.80)	0.44 (0.35, 0.53)	−0.23 (−0.39, −0.08)	0.004*	0.68 (0.55, 0.80)	0.45 (0.36, 0.54)	−0.23 (−0.38, −0.07)	0.004*
Deep white matter	0.51 (0.40, 0.61)	0.32 (0.24, 0.40)	−0.19 (−0.32, −0.06)	0.005	0.49 (0.38, 0.60)	0.33 (0.25, 0.41)	−0.16 (−0.30, −0.03)	0.016

Disease designation-specific estimates, and 95% confidence intervals, of Fisher-transformed cross-correlation among 50-years are provided, along with difference between HD subjects and controls (regardless of age, due to the lack of interaction between age and designation), and the unadjusted *p*-vales (p). * significant difference *p*_*fdr*_ < 0.05. WM, white matter.

## Discussion

Evidence from transgenic mouse models has suggested that cerebrovascular dysfunction might contribute to the early neuropathology in HD. To our knowledge, our study is the first to confirm regional impairments of cerebrovascular function *in vivo* using an externally administered CO_2_ challenge in human subjects with pre-symptomatic and early HD.

In our study, impaired CVR was predominantly found in subcortical white matter regions in HD gene-expanded individuals (Figure 2), including in the body of the corpus callosum, forceps major, superior longitudinal fasciculus, and the cingulum bundle. These correspond to white matter regions that are both known to demonstrate altered microstructural integrity (Rosas et al., 2018) and adjacent to cortical brain areas shown previously with reduced cerebral blood flow (Chen et al., 2012) and/or cortical atrophy (Rosas et al., 2002, 2008; Hobbs et al., 2010, 2011). Increased PVS burden was also found in several subcortical white matter regions including the insula, caudal middle frontal and middle temporal areas (Chan et al., 2020b) in HD. Impaired cerebrovascular function and increased PVS burden were associated with each other in cortical areas also showing reduced cerebral blood flow. These findings, together, suggest that cerebrovascular dysfunction is both early and significant in HD (Brown et al., 2018). Furthermore, they suggest that alterations in cerebrovascular function could potentially independently contribute to the neuropathology of HD.

This was supported by the presence of mutant huntingtin (mHtt) (MacDonald et al., 1993) not only in neurons but in all major components of the neurovascular unit including the basal lamina, endothelial cell, pericytes, smooth muscle cells (Lin et al., 2013; Drouin-Ouellet et al., 2015), astrocytes, oligodendrocytes, and microglia (Jansen et al., 2017). Chronic mHtt expression appears to not only alter the neurovasculature by the upregulation of WNT signaling (Lim et al., 2017) but also by affecting the expression and activity of endothelial nitric oxide (Deckel and Duffy, 2000), a vasoactive agent which could affect CVR. Disrupted cerebrovascular function may lead to reductions in CBF and prolonged relative hypoxia and/or neurovascular coupling (Kisler et al., 2017), which could then result in an increased PVS burden (Brown et al., 2018). This is consistent with our observation in HD gene-expanded subjects that greater dilated PVS load appeared to overlap with and occur along the periphery of the areas showing greater delays in cerebrovascular response (Supplementary Figure 1). Increases in PVS load have been associated with neuroinflammation, pericyte loss and breakdown of the blood-brain barrier (Sweeney et al., 2018; Nation et al., 2019), further exacerbating pathological cascades. Delayed cerebrovascular responses (Marstrand et al., 2002) or negative BOLD responses (Sam et al., 2016) to hypercapnic challenge have been previously reported in brain areas with white matter hyperintensities, compared to normal appearing white matter by as much as more than a year (Sam et al., 2016). Our finding of these changes including during the motor pre-manifest period supports that these alterations could influence not only the neuropathology of HD but contribute to clinical symptoms, including early cognitive deficits (Brown et al., 2018).

Taken together, our results support an important role for altered CVR in the pathophysiology of HD and support future longitudinal studies using CVR challenge to evaluate disease onset and/or disease progression.

### Limitations of This Study

Since this is the first study to evaluate CVR with externally administered CO_2_ to HD patients, we limited our study of gene-expanded subjects who were either motor pre-manifest or in early stages of HD. Therefore, our study is limited by a relatively small sample size and with the exclusion of more advanced patients who might have also demonstrated more profound alterations. Another limitation is the age of the HD subjects and healthy controls was significantly different; this could influence the interpretation of the differences in CVR between these two subject groups. In order to address this, we included age as a regressor of no interest to correct for the potential contribution of aging in the group analyses. Increased arterial blood pressure resulting from moderate hypercapnia can be a confound in the CVR assessment. Although we limited the mild hypercapnic condition to 4–8 mmHg above the subject’s resting P_*ET*_CO_2_, it would be ideal to continuously measure and monitor the blood pressure changes while the subjects are under hypercapnic challenge which we did not incorporate in the current study. Variations stemming from gas delivery apparatus, design of the breathing circuit, or of the protocol, to the analysis of the imaging data in CVR MRI studies using externally administered CO_2_ challenge have been reported previously (Fierstra et al., 2013; Liu et al., 2019; Chan et al., 2020a). These may be due in part to the level of the complexity of the set-up of the CO_2_ challenge, varying tolerability in different subject or patient populations, variability in the physiological responses, in addition to differences in analytic approaches used to measure the response. It would be ideal to have a standardized vasoactive stimulus available that could be applied in different settings and in different subject/patient populations; this should be an important consideration in future studies.

## Conclusion

Our preliminary findings support that alterations in neurovascular function occur in HD and may be an important, not as yet considered, independent contributor to early neuropathology and clinical symptoms in HD.

## Data Availability Statement

The original contributions presented in the study are included in the article/Supplementary Material, further inquiries can be directed to the corresponding author.

## Ethics Statement

The studies involving human participants were reviewed and approved by MGH Human Research Committee. The patients/participants provided their written informed consent to participate in this study.

## Author Contributions

SC and HR conceived and designed the study. SC and HR performed the experiments. SC and NM analyzed the data. SC, NM, KK, SH, and HR interpreted results of the experiments. SC, NM, and HR edited and revised the manuscript. All authors reviewed and approved the final version of manuscript.

## Conflict of Interest

The authors declare that the research was conducted in the absence of any commercial or financial relationships that could be construed as a potential conflict of interest.

## Footnotes

1 http://afni.nimh.nih.gov

2 http://surfer.nmr.mgh.harvard.edu

3 https://www.R-project.org

## Supplementary Material

Supplementary Figure 1 Maps of delayed cerebrovascular responses to hypercapnic challenge superimposed on T2-T1 ratio maps for individual subjects. Warm colors represent long delay response while cold colors represent short delay. Multiple comparisons are corrected using clustering at an overall *p* < 0.05.
