# Supplementary material for: Impaired Cerebrovascular Reactivity in Huntington’s Disease
Source: Front Physiol. 2021 Jul 21;12:663898. doi: 10.3389/fphys.2021.663898 (PMC8334185; doi:10.3389/fphys.2021.663898)
Supplement: Supplementary Figure 1 — Maps of delayed cerebrovascular responses to hypercapnic challenge superimposed on T2-T1 ratio maps for individual subjects. Warm colors represent long delay response while cold colors represent short delay. Multiple comparisons are corrected using clustering at an overall p < 0.05. [file Data_Sheet_1.DOCX]

Supplementary Material

# Supplementary Figures


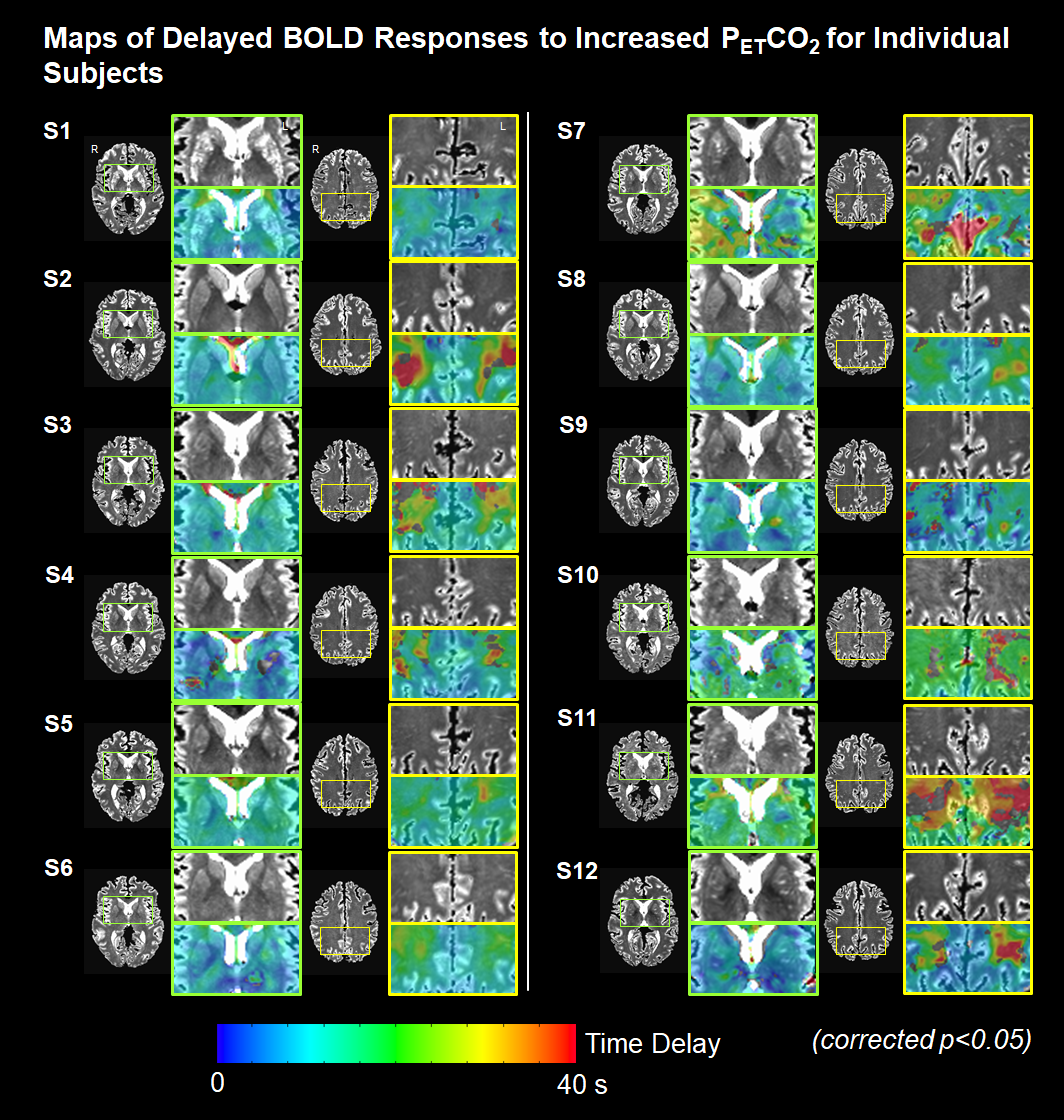


**Supplementary Figure 1.** Maps of delayed cerebrovascular responses to hypercapnic challenge superimposed on T2-T1 ratio maps for individual subjects. Warm colors represent long delay response while cold colors represent short delay. Multiple comparisons are corrected using clustering at an overall p<0.05.
